# Supplementary material for: Oxoglutarate Carrier Inhibition Reduced Melanoma Growth and Invasion by Reducing ATP Production
Source: Pharmaceutics. 2020 Nov 23;12(11):1128. doi: 10.3390/pharmaceutics12111128 (PMC7700517; doi:10.3390/pharmaceutics12111128)
Supplement: Supplementary file 1 [file pharmaceutics-12-01128-s001.pdf]

pharmaceutics

Supplementary Materials: Oxoglutarate Carrier Inhibition Abrogated Growth and Invasion by Reduction of ATP Level in Melanoma

Jae-Seon Lee, Jiwon Choi, Seon-Hyeong Lee, Joon Hee Kang, Ji Sun Ha, Hee Yeon Kim, Hyonchol Jang, Jong In Yook and Soo-Youl Kim

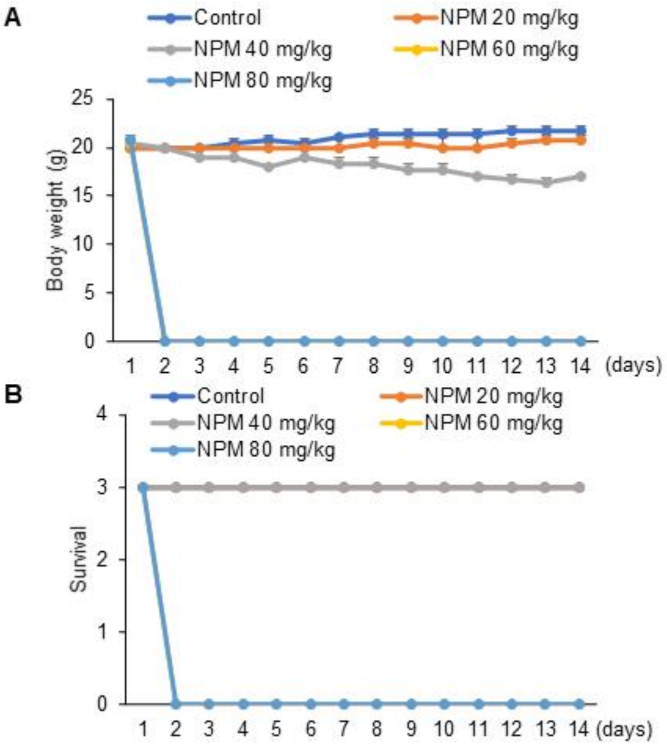

C

Cell line	
Mice	BALB/c (Orient)
N (Head)	3
Drug delivery	PO/ 5days/1week
Treatment on/off	On (5) Off (2)
Week	2
Drug dose	NPM 20, 40, 60 and 80 mg/kg
Drug vehicle	5 % DMSO +<br>10 % Kolliphor +<br>85 % PBS

Figure S1. The exploration of maximum tolerable dose (MTD) test was performed to examine the acute toxicity of NPM in animals. (**A**) Body weight of mice was measured after NPM treatment. (**B**) Survival rate of mice measured after NPM treatment. (**C**) MTD condition table.

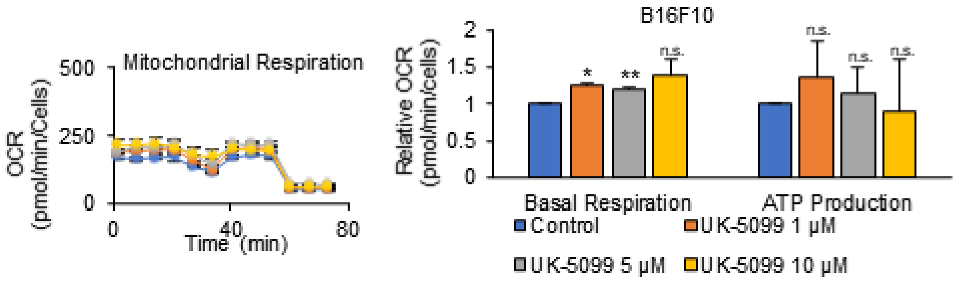

Figure S2. The cell mito stress test was performed to examine the effect of UK-5099 as mitochondria pyruvate carrier inhibitor. OCR and ATP production were measured in B16F10 cells treated with indicated concentration of UK-5099 for 24 h using a Seahorse XFe96 analyzer. Data represent the mean and standard deviation of three independent experiments. * $p < 0.05$ and ** $p < 0.01$ compared with the vehicle control.

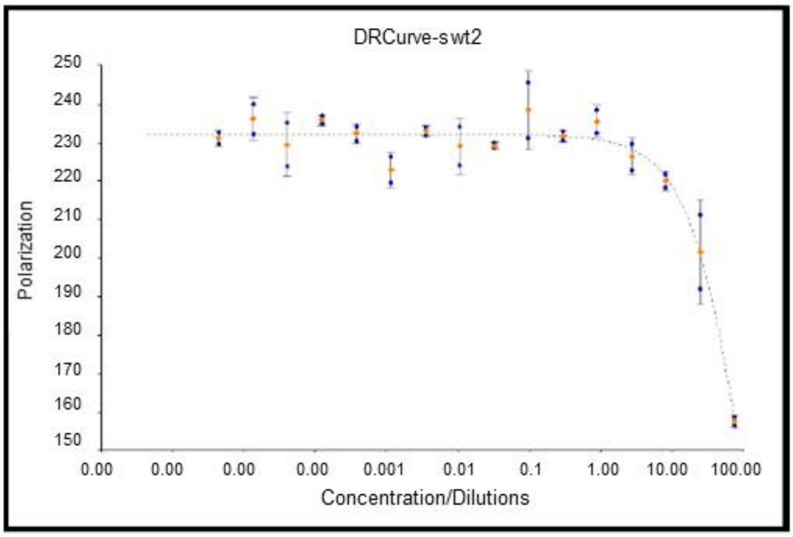

curve	Y formula	Y	X
DRCurve-s w2	IC50	116	>78.750 m M

Figure S3. The hERG K+ channel Binding assay of NPM was performed to examine the cardiac toxicity. We consider the IC50 value of 10 μM as safety bottom line. The IC50 value is higher than 78 μM and is considered safe against cardiac toxicity caused by hERG K+ CHANNEL inhibiting.
